# 3D analysis of the distal ulna with regard to the design of a new ulnar head prosthesis

**DOI:** 10.1186/s12891-022-05480-w

**Published:** 2022-06-02

**Authors:** Pascal Raffael Furrer, Ladislav Nagy, Lisa Reissner, Andreas Schweizer

**Affiliations:** Department of Orthopedics, Balgrist University Hospital, University of Zürich, Forchstrasse 340, 8008 Zürich, Switzerland

**Keywords:** Distal ulnar anatomy, Ulnar rotation, Ulnar offset, Ulnar head size, Distal ulnar shaft size, Ulnar head arthroplasty

## Abstract

**Study design:**

A retrospective, single center, data analysis.

**Objective:**

Persistent pain and instability are common complications after distal ulnar head arthroplasty. One main reason may be the insufficient representation of the anatomical structures with the prosthesis. Some anatomical structures are neglected such as the ulnar head offset and the ulnar torsion which consequently influences the wrist biomechanics.

**Methods:**

CT scans of the ulnae of forty healthy and asymptomatic patients were analyzed in a three-dimensional surface calculation program. In the best fit principle, cylinders were fitted into the medullary canal of the distal ulna and the ulnar head to determine their size. The distance between the central axes of the two cylinders was measured, which corresponds to the ulnar offset, and also their rotational orientation was measured, which corresponds to the ulnar torsion.

**Results:**

The mean medullary canal diameter was 5.8 mm (±0.8), and the ulnar head diameter was 15.8 mm (±1.5). The distance between the two cylinder axes was 3.89 mm (±0.78). The orientation of this offset was at an average of 8.63° (±15.28) of supination, reaching from 23° pronation to 32° supination.

**Conclusion:**

With these findings, a novel ulnar head prosthesis should have different available stem and head sizes but also have an existing but variable offset between these two elements. A preoperative three-dimensional analysis is due to the high variation of offset orientation highly recommended. These findings might help to better represent the patients natural wrist anatomy in the case of an ulnar head arthroplasty.

**Level of Evidence:**

III.

## Introduction

**Fig. 1:**
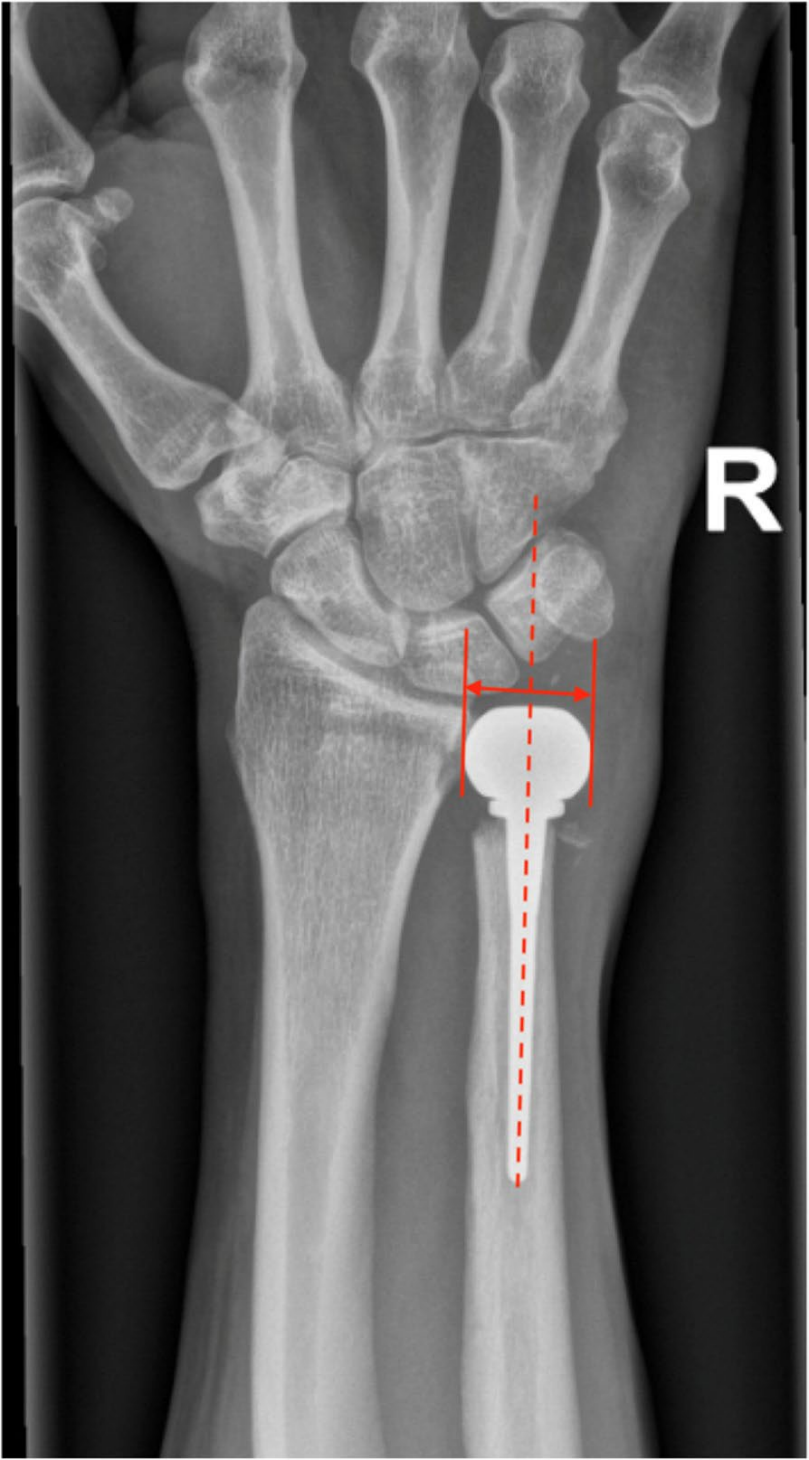
Example of a conventional ulnar head prosthesis of a 55yo male due to posttraumatic DRUJ arthritis

The distal radioulnar joint (DRUJ) has a biomechanically complex mechanism with many interacting anatomical structures [1–3]. Various causes may lead to distal radioulnar pain and dysfunction, one being DRUJ degeneration [2–4]. One of the treatment options is an arthroplasty of the distal ulnar head [5]. Although many authors have reported almost perfect postoperative outcomes over time, ulnar arthroplasty does have many failures, and the patients are not always satisfied with any type of prosthesis [6–8]. The leading reasons for suboptimal outcomes are persistent pain, prosthesis instability and a restricted range of motion [7, 9, 10]. Complication rates needing revision surgery are reported in up to 29% of cases [7, 11, 12]. In most of the literature on this topic, the inventors of the prostheses are involved, which might introduce a certain bias of positive outcome of their data [10, 12]. The authors of this paper, experienced hand surgeons, cannot confirm the good results presented by the inventors of the prostheses, and in the cases of limited range of motion and instability, suspect that an insufficient representation of the osseous anatomy is one improvable component. Especially for the total ulnar head prosthesis, the main stabilizer, the triangular fibrocartilage complex (TFC), which is responsible for > 50% of the stability of the DRUJ, is resected [13]. A biomechanical analysis has shown that adding offset to the prosthesis can introduce significant stability for the DRUJ [13]. Looking at the previous prostheses, no offset was implemented with the overlaying axis of the shaft and head (Fig. 1). Other previous studies have analyzed the anatomy of the proximal and distal radioulnar joint, as well as the osseous anatomy of the distal ulna [14–16]. The recommendation for the development of a prosthesis with a non-in-line laying shaft and head has therefore been given before [16]. Although measured previously, the parameters used, do not seem to be ideal for the development of a new ulnar head prosthesis.

The aim of this study was to describe a method to measure the necessary parameters of the distal ulna reproducibly and to clarify its shape to lay a foundation for further prosthetic designs, which better respects the physiological anatomy. We think the head and shaft size and their relative positioning to one another is a very important factor influencing the postoperative restriction of movement and DRUJ stability, therefore its outcome.

## Materials and methods

Computed tomography (CT) (slice thickness 1 mm; 120 kV; Philips Brilliance 40 CT, Philips Healthcare, The Netherlands) data of the forearm of 40 patients out of an anonymized patient pool with no pathology were included in this study and assessed. These scans were made for patients with contralateral forearm fractures to use the nonaffected side as a healthy template for 3D-guided, patient-specific osteotomies [17]. The CT data were segmented using commercial segmentation software Mimics (Materialise, Leuven, Be) to create 3D surface models of the forearm and were imported to our in-house developed software CASPA (Balgrist Card AG, Zurich, Switzerland), which enables us to use its CAD functions. Approval from the local ethical committee (BASEC-Nr. Req-2021–00691) and informed patient consent were obtained.

Four hand surgery-trained physicians measured each ulna as described below, and its conformity was verified.

### 3D measuring method

**Fig. 2:**
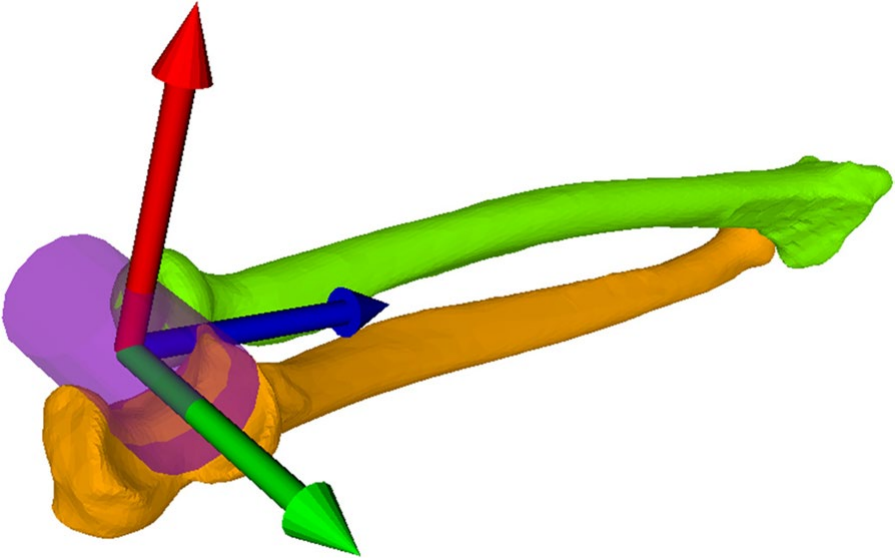
The cylinder was fitted into the ulnar trochlea. The coordinate system was aligned with the green arrow (y-axis) overlaying the humeral cylinder axis and is perpendicular to the ulnar axis. The blue arrow (z-axis) was set in the direction of the ulna, although its alignment does not have an influence on the here presented measurements

A cylinder with its corresponding coordinate system (cylinder axis = y-axis) was fitted in the proximal ulna. The y-axis was perpendicular to the ulnar shaft axis. Then the cylinder was fitted into the ulnar trochlea. A previous anatomical study has shown a very constant radial notch at the proximal ulna within a limit of +/− 2°, therefore the proximal radial head was used for rotational orientation around the z-axis [14]. This type of coordinate system setup has already been used in other studies before and it allows measuring pro- and supination as well as torsion of the Ulna [18] (Fig. 2).

**Fig. 3:**
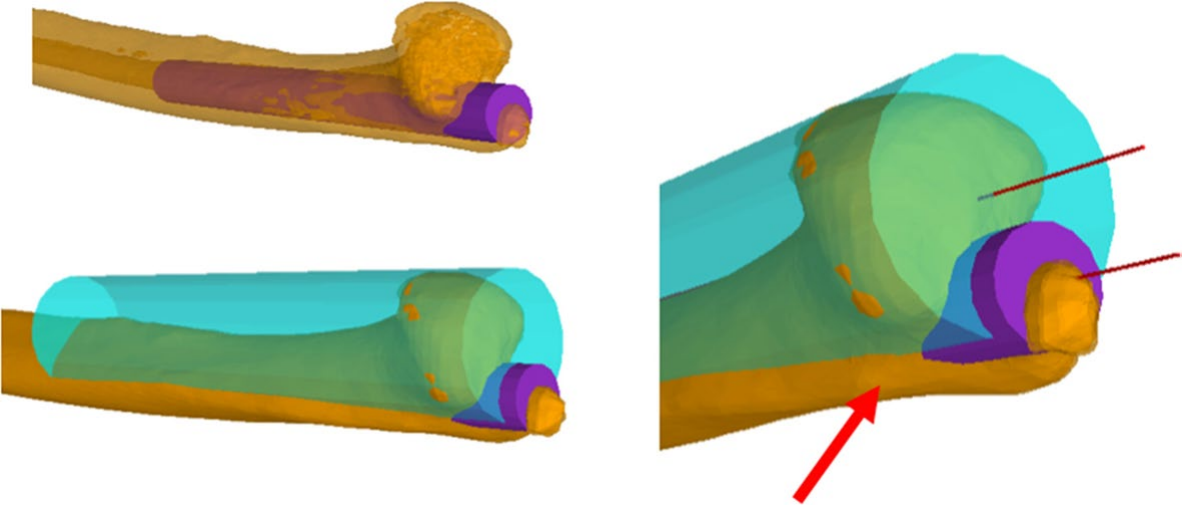
Top left: The shaft cylinder is fitted into the surface reconstructed ulna, also seeing the inner part when the bone is set as translucent. Bottom left: The head cylinder is always parallel positioned and is fitted to barely cover the ulnar hear. Right: Best fit of the ulnar head cylinder shows non-congruence with the medial ulnar wall (red arrow). This leads to difference in value of the head size and offset compared to previous analyzes

**Fig. 4:**
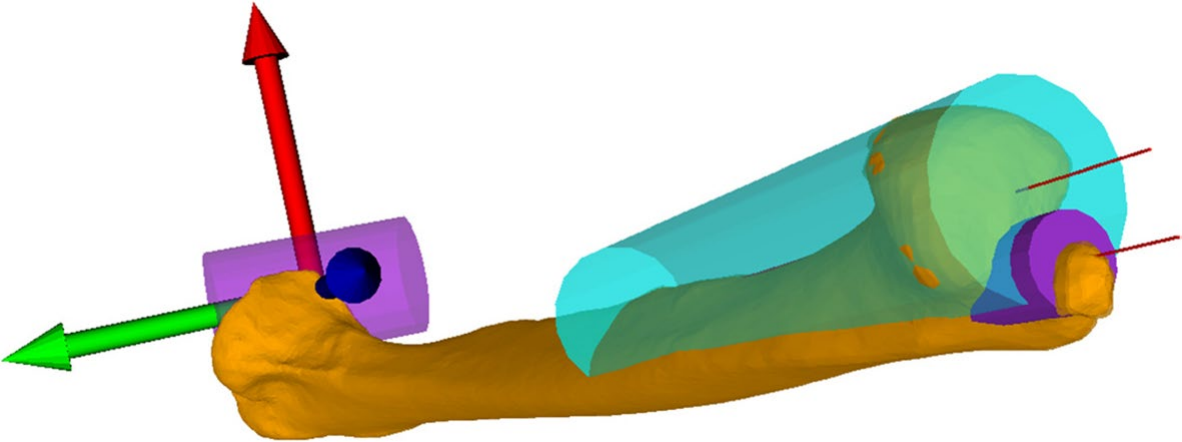
The center axes are shown and its distance was measured resulting in the ulna offset distance. The ulnar rotation was measured with a plane aligned with the coordinate system, zero being parallel to the red arrow (x-axis). This plane was then pulled into the shaft center, which was also defined as the rotation axis. From there the plane was rotated around the axis resulting in the amount of pro- and supination

Second, an eight-centimeter-long cylinder was fitted into the distal ulnar shaft, guiding the shaft size and alignment by the method of best fit measuring the biggest possible intramedullary canal without affecting the cortical bone. Then, a cylinder with a parallel axis was fitted onto the ulnar head, guiding the size of the ulnar head in the same manner as for the shaft (Fig. 3). The distance between the two cylinder axes was measured, and its relative rotation, pro- and supination, was measured using the initial set coordinate system as a reference (Fig. 4). This rotational orientation of the offset represents the ulnar torsion. 0° of rotation was set parallel to the x-axis.

### Statistics

Normal distribution of all data was evaluated with the Kolmogorov–Smirnov test, and correlations between the measured variables of the distal ulna were calculated with linear regression. Measurement agreement between the four readers was calculated with a 2-way mixed absolute interclass correlation (ICC). Linear regression was used to determine the dependence of the head and shaft size. Data are presented as the mean ± standard deviation.

## Results

All 40 included forearms were used for the calculation and analysis.

### Intraclass correlation (ICC)

All four measurements showed significant and good to very good ICC between the readers. The ICC for the offset was 0.817, for the ulnar rotation 0.895, for the head size 0.927 and the shaft size 0.877.

### Sizing

**Fig. 5:**
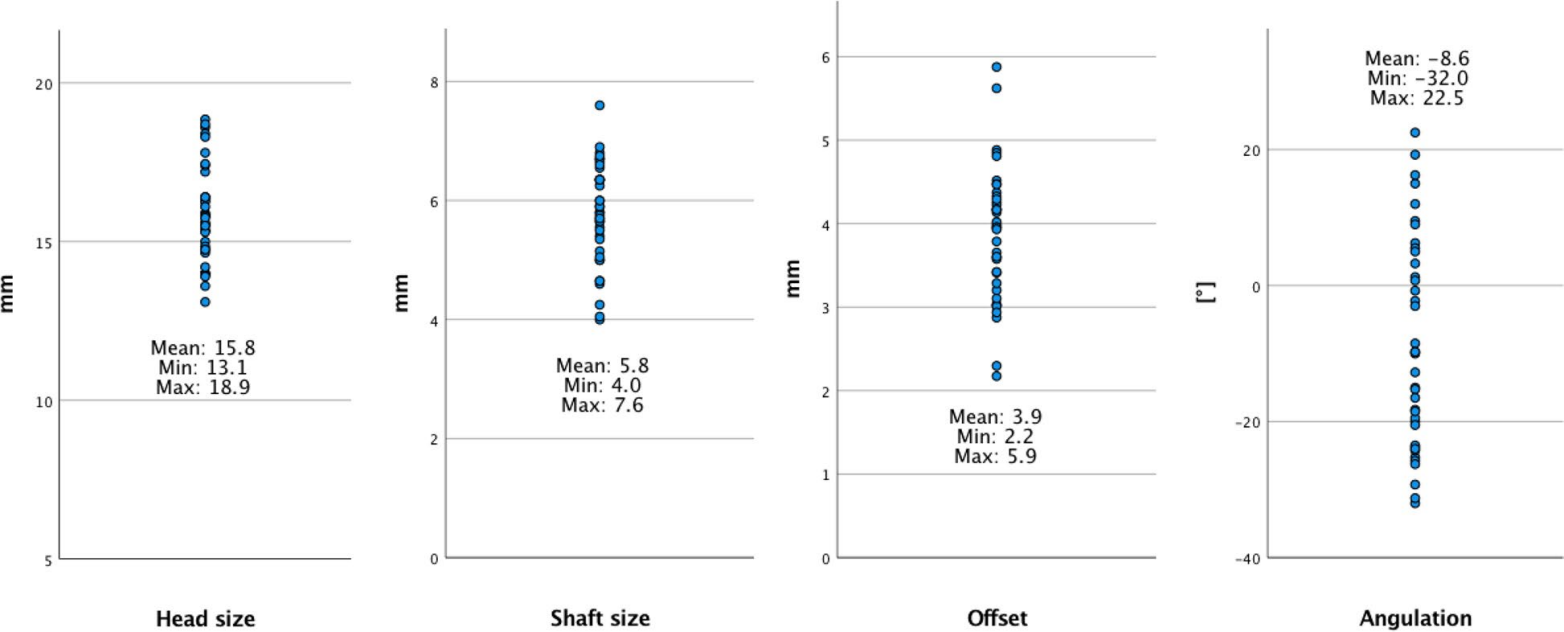
Distribution of offset, ulna rotation, shaft size and head size, with the given mean, lowest and highest value. Angulation: positive values are representing pronation, negative values are representing supination

The ulnar head size was 15.8 mm (±1.5), and the ulnar distal shaft cylinder size was 5.8 mm (±0.83). The offset distance between the centers of the two cylinders was 3.89 mm (±0.78). The rotation was at an average 8.63° (±15.28) in the supinated position with a wide standard deviation. The rotation was widely distributed, reaching over 20° pronation to over 30° supination (Fig. 5).

A positive linear correlation between the head and shaft size was observed (R^2^ = 0.387, *p* = 0.001). No correlation was seen between the other measured variables.

## Discussion

The main findings of this study are that there is an offset of 3.9 mm between the axis of the ulnar head and the ulnar shaft and that its rotational orientation has a spread of over 50°. These parameters, among others, must be taken into account for a new prosthesis design on the one hand and for implantation and preoperative assessment on the other.

A previous study analyzed CT scans of distal ulnae and concluded that a modular prosthesis with an offset would be best to achieve the correct fit [16]. However, for the ulnar head, they measured the entire diameter of the distal ulna, which as we observed does not fit as accurately as if one put a cylinder with the best fit to only the articular surface of the ulnar head (red arrow, Fig. 2). This leads to an overestimation of the ulnar head size and underestimation of the offset. They have measured a mean offset of 2.5 mm, which is only about half of the offset that should be implemented in a new prosthesis according to our calculations. Torsion of the ulna was not evaluated in this study, which is decisive for the implantation direction of a prosthesis.

A similar study, made with regards to general surgical implications, focused on the rotation of the radius and the ulnae, which is very helpful as an addition to our study, yet it was not sufficient for a prosthesis design, since no information about the head and shaft sizing is given [15]. Interestingly, where our study found a rather supinated rotation of the ulnae, their sample had an average pronation of 8.4° in their ulna. Though more importantly, the confirmation of a wide range of ulnar torsion, from 50° pronation to 22° supination. The absolute values of pro- and supination may not be comparable since they used a different measuring method, but the variations among the specimens is approximately the same and supports our findings. This highlights the necessity of our data and the need for correct rotational placement of the prosthesis. We are therefore convinced that a preoperative CT scan of the whole forearm will be unavoidable to obtain the correct rotational implantation of the prosthesis with its offset, since distal landmarks, for example, the ulnar styloid, can only be used as a constant landmark to a very limited extent [19].

As mentioned before the offset distance is highly important, which has been shown recently in a biomechanical analysis to be a major factor for DRUJ stabilization [16]. The distance between the center of the shaft and the ulnar head is essential to retain the maximum possible residual stability, since the main source of stability, the TFC, is currently being resected during total head arthroplasty [6]. With resection of the TFC, the intraosseus membrane with its central band and its distal oblique bundle, as well as the distal interosseous ligaments, are of particular importance. Only with the correct amount of offset are these ligamentous structures placed in proper tension to give the necessary amount of stability. The interosseous ligament complex is also responsible for axial translation, so loosening of this firm structure can lead to an ulna plus variance and thus ulnar sided wrist pain [20]. In previous prostheses, no offset was implemented with the overlaying axis of the shaft and head (Fig. 1). Only one partial head prosthesis (First Choice DRUJ System, Integra, Austin, TX) had a very small offset, which is not sufficient according to our findings. Therefore, without the correct offset, instability and ulnar-sided wrist pain are predictable.

It will be especially important to combine the parameters found here for the sizes and the rotational direction of the offset when operatively implemented. Rotational malplacement (around the z-axis) of an offset prosthesis would probably lead to poor tension conditions, such as over tension in pronation and under tension in supination or vice versa, leading to pain and functional restriction. Therefore, not only will the prosthetic components need to be adapted but also the preoperative evaluation of each patient and the rotational method of installation needs to be calculated.

There are definitely limitations to this study, first the patient population of 40 patients. A larger population of patients might have changed the overall values, but we do not expect this to be the case, and if so, only minimally. Another limitation is the retrospective study design. This leads to some inability to do certain things that could have been adjusted in a prospective study design. However, we used healthy wrists in mixed epidemiology, where a prospective study design could not have helped us.

On the other hand, we focused more on the alignment and size of the different osseous elements, less on their individual shape. However, for example a curvature of the distal ulna cannot be addressed with a standard prosthesis anyway and has to be measured individually in a case out of norm. Also, the documentation of the different shapes of the head and radial sigmoid notch is already given and may be taken from the literature.

## Conclusion

A reliable three-dimensional method was evaluated to measure the parameters necessary for the distal ulnar prosthesis design. A correlation was only seen between the distal ulnar head and shaft size. A very important variable was the offset between the axis of the ulnar shaft and the ulnar head, if neglected, is assumed to be causable for postoperative instability, ulnar sided wrist pain and limited range of motion. Interestingly, the scatter of the rotation value was very high, supporting the need for three-dimensional preoperative planning.

## Data Availability

The datasets used and/or analyzed during the current study are available from the corresponding author on reasonable request.
